# Case Report: Unplanned pregnancy during treatment with inebilizumab in patients with AQP4-IgG-seropositive NMOSD: report of two cases

**DOI:** 10.3389/fimmu.2026.1953059

**Published:** 2026-09-24

**Authors:** Wenqin Luo, Fenglin Shi, Zhu Xu, Yunli Yu, Dian He

**Affiliations:** Department of Neurology, Affiliated Hospital of Guizhou Medical University, Guiyang, Guizhou, China

**Keywords:** B-cell depletion, case report, inebilizumab, neuromyelitis optica spectrum disorder, pregnancy

## Abstract

Neuromyelitis optica spectrum disorder (NMOSD) predominantly affects women and has a median age at onset of 40 years, meaning that many patients are of childbearing age. However, management of pregnant women with NMOSD remains extremely challenging. We report two patients with NMOSD who had unplanned pregnancies during inebilizumab (INE) treatment and successful deliveries. These cases provide preliminary clinical information regarding maternal disease control and pregnancy outcomes following INE exposure.

## 1 Introduction

Neuromyelitis optica spectrum disorder (NMOSD) is a relapsing autoimmune disease of the central nervous system (1) that frequently causes severe neurological deficits (2). Pathogenic antibodies against aquaporin 4 (AQP4-IgG) are detected in more than 80% of patients with NMOSD (3). NMOSD predominantly affects women and has a median age at onset of 40 years (4). Because many patients with NMOSD are of childbearing age and wish to give birth, the management of pregnancy in such individuals has become a critical focus in clinical practice. Inebilizumab (INE) is a B-cell-depleting monoclonal antibody widely used in AQP4-IgG-seropositive NMOSD. Although a case of unplanned pregnancy during INE treatment has recently been reported (5), evidence regarding the safety of INE during pregnancy remains extremely limited. In this report, we present two cases of unplanned pregnancy in patients with NMOSD receiving INE treatment.

## 2 Case reports

### 2.1 Case 1

In 2015, a 20-year-old Chinese woman presented with vision loss in her left eye and was admitted to the Department of Neurology at the Affiliated Hospital of Guizhou Medical University. In 2013, she had experienced transient lower-limb numbness and dysuria but recovered completely without treatment. In 2014, she developed severe vision loss in her right eye (Snellen acuity, 20/200). She was admitted to the Department of Ophthalmology and diagnosed with acute optic neuritis (ON). After treatment with 1 g of prednisolone daily for 3 days, vision in the right eye remained impaired. On admission in 2015, acute ON was confirmed by contrast-enhanced magnetic resonance imaging of the left optic nerve and a positive serum AQP4-IgG cell-based assay, leading to a diagnosis of NMOSD (6). After treatment with high-dose intravenous methylprednisolone (IVMP) and a subsequent oral prednisolone taper, vision in the left eye recovered completely. She subsequently received intravenous rituximab (RTX) for relapse prevention. However, between 2015 and 2023, she experienced seven relapses, including transverse myelitis and ON. In 2023, following two bilateral ON relapses, Sjögren’s syndrome (SS) was diagnosed via positive anti-SSA antibodies and salivary gland biopsy. Following partial recovery after IVMP, intravenous immunoglobulin, and plasma exchange, the patient repeatedly underwent oral prednisolone tapers for each relapse, but refused maintenance immunosuppressive therapy (IST) for 8 years. After the last relapse in 2023, following the recommendation of the neurologists, INE maintenance therapy was initiated. The patient received two INE doses in November and December 2023, and had no relapses subsequently. Despite a strict requirement for contraception, the patient reported an unplanned pregnancy in May 2024 (five months after the last INE infusion). Therefore, the next planned INE infusion in June 2024 was canceled, and the patient was regularly monitored in a multidisciplinary outpatient clinic comprising experts from the Departments of Neurology, Obstetrics and Gynecology, and Rheumatology.

During pregnancy, the patient declined IST; her low CD19^+^ B-cell count persisted without NMOSD relapse, and she successfully delivered by cesarean section at 38 + 6 weeks on February 11, 2025. The infant weighed 2,920 g, had no birth defects, and was breastfed. The patient’s B-cell count remained low, and no B-cell depletion was observed in the newborn (**Table 1**). INE was resumed immediately postpartum. At the latest follow-up, no NMOSD relapse had occurred, and the infant was developing normally. The timeline and B-cell changes are summarized in **Figure 1**.

**Table 1:** Percentages and counts of lymphocytes in the newborn in Case 1.

Laboratory test of lymphocytes	Results	Reference range
CD3+ T cells (percentage)	58.57%	52.11%–81.55%
CD8+ T cells (percentage)	13.56%	14.19%–43.41%
CD4+ T cells (percentage)	44.55%	22.2%–50.25%
NK cells (percentage)	24.62%	6.85%–36.98%
CD19+ B cells (percentage)	15.03%	5.05%–20.45%
CD3+ T cells (count)	1814/μL	834/μL–2217/μL
CD8+ T cells (count)	420/μL	269/μL–1059/μL
CD4+ T cells (count)	1380/μL	395/μL–1264/μL
NK cells (count)	763/μL	136/μL–880/μL
CD19+ B cells (count)	466/μL	92/μL–498/μL

**Figure 1:**
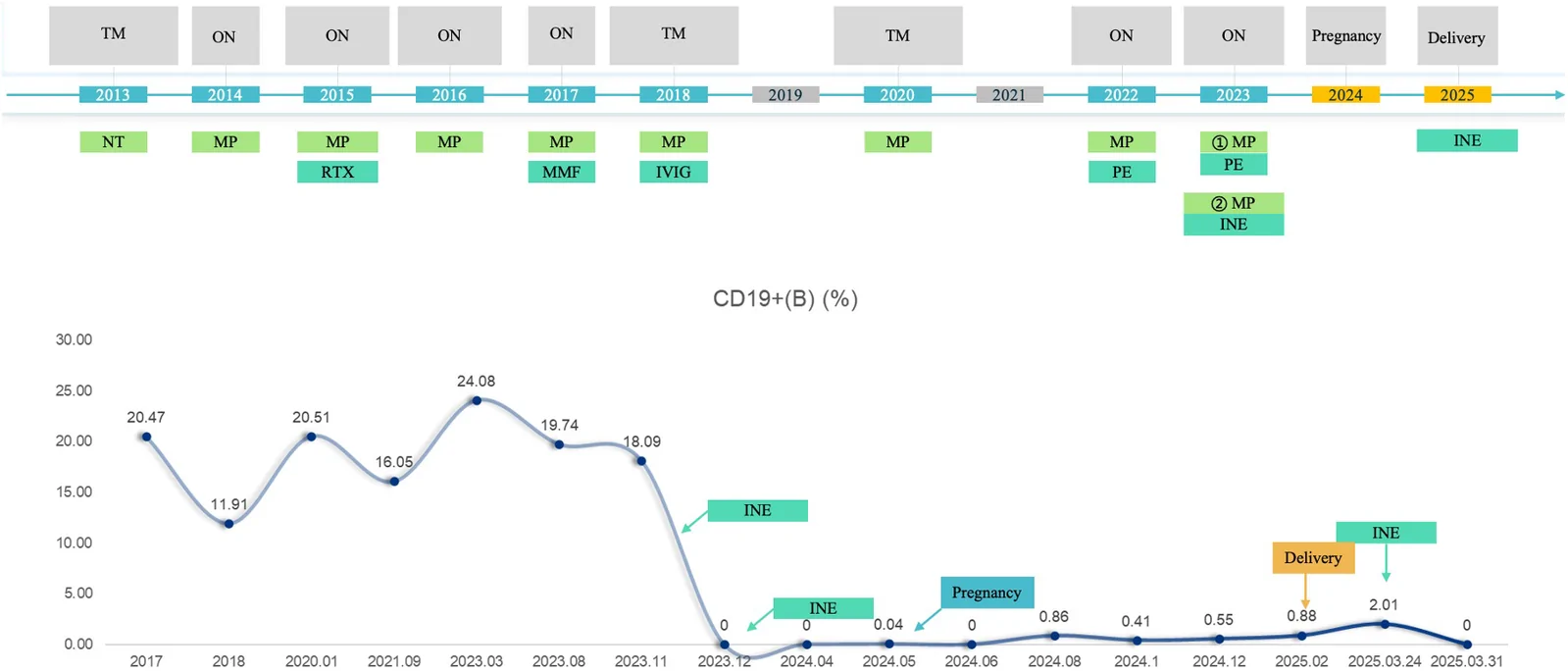
Timeline and changes in the percentage of CD 19 (+) B cells of Case 1. INE, inebilizumab; IVIG, intravenous immunoglobulin; MMF, mycophenolate mofetil; MP, methylprednisolone; NT, no therapy; ON, optic neuritis; RTX, rituximab; TM, transverse myelitis.

### 2.2 Case 2

A 26-year-old woman was admitted to the Department of Neurology with numbness of the extremities in 2018. Cervical spinal cord enhancement suggested acute transverse myelitis, and a positive serum AQP4-IgG cell-based assay confirmed NMOSD. Following complete recovery after IVMP and oral prednisolone tapering, the patient received RTX maintenance treatment. Concurrently, SS was diagnosed based on positive anti-SSA antibodies and salivary gland biopsy and was managed with hydroxychloroquine (HCQ). The patient remained relapse-free until 2020, when she was readmitted with limb numbness. Complete recovery was achieved after IVMP and intravenous immunoglobulin, followed by mycophenolate mofetil (MMF) maintenance therapy. After remaining relapse-free for 4 years, the patient self-discontinued MMF in January 2024. Two months later, she was readmitted with limb pain and numbness due to an NMOSD relapse and was treated with IVMP. Maintenance INE treatment was initiated, but the patient conceived 1 month after the third dose. Following multidisciplinary discussions among the Departments of Obstetrics and Gynecology, Neurology, and Rheumatology, HCQ and low-dose oral prednisone were used during pregnancy were indicated to manage SS and NMOSD. B-cell counts rapidly rebounded during pregnancy, but the patient remained relapse-free. She delivered via cesarean section at 38 weeks in September 2025. Because the patient could not afford INE infusions and was transitioned to MMF after delivery. No B-cell depletion occurred in the infant (**Table 2**). The timeline of events and changes in B-cell percentage in this patient are summarized in **Figure 2**. The timelines of treatment, NMOSD attacks, pregnancy, and delivery in both cases are summarized in the **Supplementary Figure 1**.

**Table 2:** Percentage and counts of lymphocytes in the newborn in Case 2.

Laboratory test of lymphocytes	Results	Reference range
CD3+ T cells (percentage)	73.71%	52.11%–81.55%
CD8+ T cells (percentage)	22.49%	14.19%–43.41%
CD4+ T cells (percentage)	48.01%	22.2%–50.25%
NK cells (percentage)	9.01%	6.85%–36.98%
CD19+ B cell (percentage)	16.58%	5.05%–20.45%
CD3+ T cell (count)	3738/μL	834/μL–2217/μL
CD8+ T cell (count)	1141/μL	269/μL–1059/μL
CD4+ T cell (count)	2435/μL	395/μL–1264/μL
NK cell (count)	457/μL	136/μL–880/μL
CD19+ B cell (count)	841/μL	92/μL–498/μL

**Figure 2:**
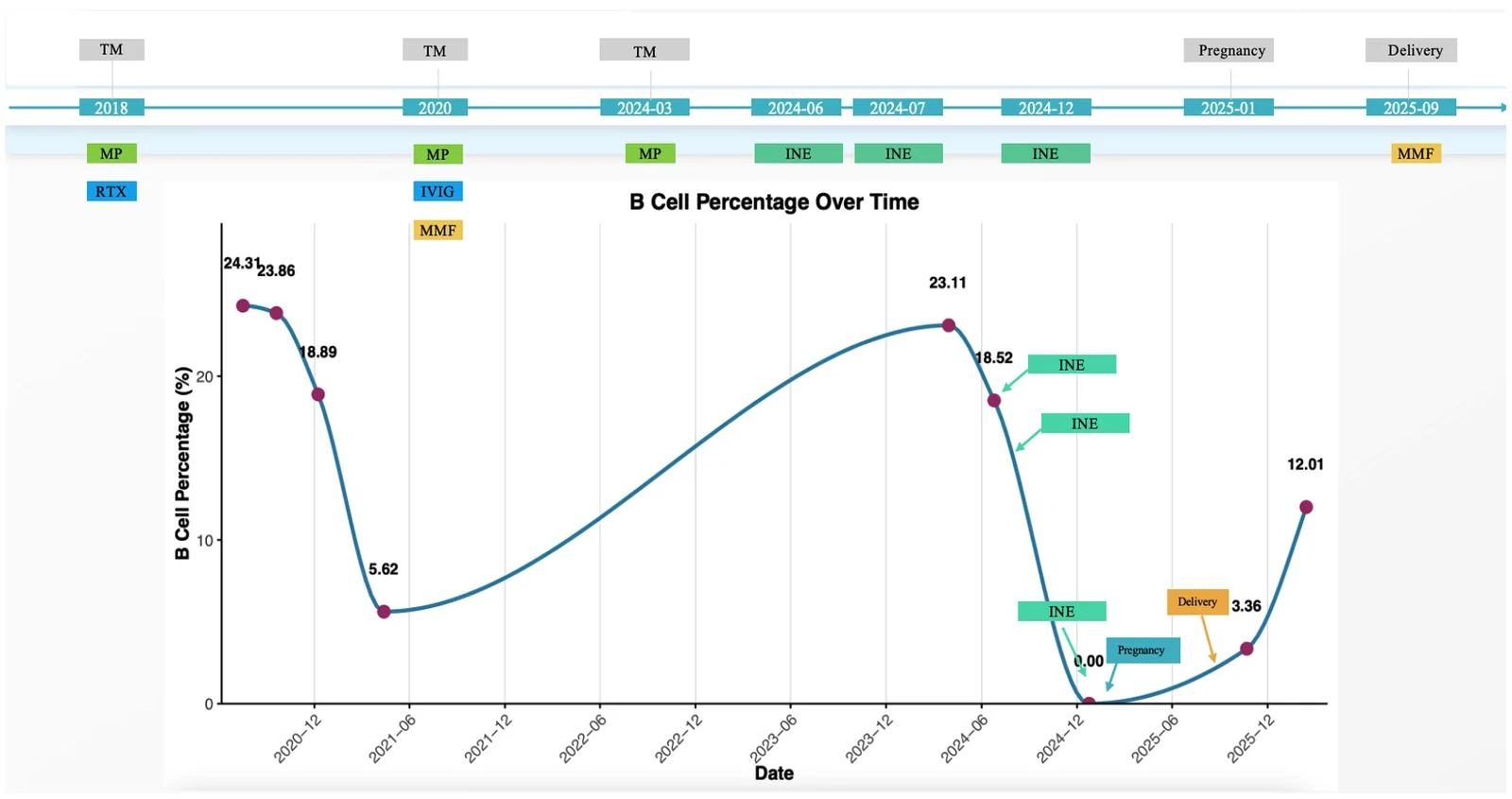
Timeline and changes in the percentage of CD 19 (+) B cells of Case 2. INE, inebilizumab; IVIG, intravenous immunoglobulin; MMF, mycophenolate mofetil; MP, methylprednisolone; ON, optic neuritis; PE, plasma exchange; RTX, rituximab; TM, transverse myelitis.

## 3 Discussion

In this report, we describe two patients with NMOSD who had unplanned pregnancies after INE treatment but had successful deliveries and remained relapse-free during pregnancy and postpartum. The infants were healthy with no birth defects. Our report provides preliminary clinical insights into the safety and potential application of INE in pregnant women with NMOSD.

Pregnancies in patients with NMOSD are considered high risk. In contrast to multiple sclerosis, NMOSD disease activity does not subside during pregnancy, and the Expanded Disability Status Scale score increases significantly during pregnancy (7–9). A higher risk of relapse was observed in patients who did not undergo IST during pregnancy (8). Mechanistically, maternal AQP4-IgG can passively cross the placenta and bind to AQP4 expressed on placental syncytiotrophoblasts (10), resulting in a higher risk of obstetric complications. However, a recent systematic review found no statistically significant differences in premature deliveries, abortions, preeclampsia, or neonatal complications between patients with NMOSD and the general population (8). *In utero* exposure to maternal AQP4-IgG resulted in brain dysfunction in mouse offspring (11), although the clinical relevance of these findings in humans remains uncertain. Despite the debate on the impact of NMOSD on offspring and pregnancy outcomes, disease control during pregnancy is considered beneficial for patients with NMOSD.

CD19^+^ B-cell levels correlate with relapse risk in patients receiving B-cell depletion therapy (12–14). In patients treated with RTX, a modified and individualized re-dosing strategy based on B-cell levels has been suggested instead of re-dosing every 6 months (15, 16). INE is also a B-cell-depleting agent, so the modified re-dosing strategy employed for RTX may be extrapolated to INE after necessary validation. Case 1 had very low B-cell levels (less than 1%) without prenatal IST, which may have contributed to the lack of relapse during pregnancy and postpartum. Case 2 experienced a rapid restoration of B-cell counts but was treated with HCQ, which may have decreased the relapse risk during and after pregnancy.

Considering the severe neurological dysfunction resulting from NMOSD attacks, appropriate IST during pregnancy may be important for maintaining disease control. However, concerns over obstetric or fetal outcomes limited IST use during pregnancy, leaving pregnant patients prone to postpartum relapse (17). The safety of RTX in pregnancy remains controversial. RTX is generally not recommended during pregnancy by the French Multiple Sclerosis Society (18), although another review considers it relatively safe during pregnancy (19).

Placental crossing of B-cell-depleting monoclonal antibodies from the mother may cause transient neonatal B-cell depletion (20). Based on this, two different approaches have been proposed for the timing of B-cell-depleting therapy around pregnancy. First, a recent expert review suggested that re-dosing with B-cell depletion therapy may be considered in the late first trimester (gestational week 12), especially in patients with active disease within the 2 years before pregnancy (21). Transplacental IgG transfer is minimal early in pregnancy and increases markedly with advancing gestation, particularly during the last four gestational weeks (21), it was reported that fetal IgG concentrations are only approximately 5%–10% of maternal concentrations at 22 weeks of gestation (22). The terminal half-life of INE is approximately 18 days; therefore, if the last INE infusion occurs at the late first trimester, maternal INE concentrations would be expected to be substantially reduced after approximately five half-lives (90 days, gestational week 25). Second, some guidelines recommend avoiding monoclonal antibody use during pregnancy. A recent consensus suggested that pregnancy can be attempted one month after RTX infusion, although data on the use of INE during pregnancy are very limited, INE, like RTX, has prolonged biological activity and may be discontinued 1 month before conception (23). Other guidelines recommend contraception for the first three months after the last RTX infusion (18, 24, 25). Because transplacental IgG transfer is limited early in pregnancy and increases from the second trimester onward (20), fetal exposure is expected to be lower when maternal drug concentrations have declined substantially before this period. In both strategies, fetal exposure to therapeutic antibodies and the risk of neonatal lymphocytopenia are theoretically low. Nevertheless, we strongly recommend monitoring lymphocyte and B-cell counts in exposed newborns. It should be noted that the recommendations above are all based on the clinical data of RTX administration in pregnant women. Whether these recommendations can be directly applied to the infusion of INE in pregnant women requires further observations. Both our patients had NMOSD relapses within 1 year before pregnancy; however, because reports of INE treatment in pregnant women remain limited, INE re-dosing was not performed throughout pregnancy in either case.

High rates of cesarean delivery have been reported in patients with NMOSD (26, 27). The French Multiple Sclerosis Society recommended that the indications for cesarean delivery used in the general population also apply to women with NMOSD, regardless of the level of disability (18). However, a national survey of cesarean delivery rate in China reported an overall cesarean section rate of 43.5% (28). Studies of Chinese populations have also reported associations between intolerance of uncertainty or fear of childbirth and cesarean section preference or mode of delivery (29, 30); such anxiety may be more prominent in women with NMOSD. In our report, neither patient had spasticity, paraplegia, or perineal sensory loss, and neither had standard obstetric indications for cesarean delivery; however, both requested cesarean delivery because of fear related to NMOSD. There is no evidence to suggest that the method of delivery may affects NMOSD activity (31). Therefore, following evaluation by obstetricians and neurologists, patient preference for cesarean del may also be considered.

Although therapeutic monoclonal antibodies are secreted into breast milk, they degrade in the infant gut and are unlikely to influence infant immune function (32–34). Thus, breastfeeding was recommended in case 1.

This study has several limitations. First, the study is based on just two cases, so its conclusions cannot be generalized to the broader population. Second, the follow-up duration of the study was relatively short, limiting assessment of the long-term safety of INE treatment in pregnant patients.

In conclusion, we have described two cases of NMOSD with unplanned pregnancy during treatment with INE. Both patients had successful deliveries, and no B-cell depletion was observed in the infants. These observations provide preliminary clinical information regarding INE exposure during pregnancy. However, larger-scale observational cohort studies are required to assess the safety of INE exposure during pregnancy and its effects on pregnancy outcomes.

## Data Availability

The raw data supporting the conclusions of this article will be made available by the authors, without undue reservation.

## Supplementary material

Supplementary Figure 1 Treatment timeline and clinical events in both cases. Only treatments for NMOSD relapse prevention are displayed. INE, inebilizumab; MMF, mycophenolate mofetil; NT, no therapy; RTX, rituximab.
